# Soil Fertility and Phosphorus Leaching in Irrigated Calcareous Soils of the Mediterranean Region

**DOI:** 10.1007/s10661-023-11901-7

**Published:** 2023-10-26

**Authors:** Carlos Ortiz, Silvia Pierotti, M. Gabriela Molina, Àngela D. Bosch-Serra

**Affiliations:** 1 Department of Chemistry, Physics, Environmental Sciences and Soil, University of Lleida, Avda Alcalde Rovira Roure 191, E-25198 Lleida, Spain; 2 DACC, Department of Climate Action, Food and Rural Agenda, Generalitat de Catalunya, Avda Alcalde Rovira Roure 191, E–25198 , Lleida, Spain; 3 Cátedras de Bioestadística I y II. Facultad de Ciencias Exactas, Físicas y Naturales, Universidad Nacional de Córdoba, Córdoba, Argentina

**Keywords:** Available phosphorus, Farm-scale survey, Heavy metals, Mineral fertilizer, Organic fertilizer

## Abstract

To ensure soil quality and soil health, it is necessary to improve fertilization practices while minimizing environmental impacts. The aim of this study was to record the state of the art on soil fertility related to fertilization management (organic and/or mineral) and to detect environmental challenges in highly productive fields. A soil survey was set up in a new irrigated area (*c*. 20 years old), in the north-eastern part of Spain, which is mainly devoted to double annual crop rotations of cereals and maize. The area also supports an important animal rearing activity. The survey covered 733 ha of calcareous soils, owned by 35 farmers. At each farm, fertilization management was recorded, and soil was analyzed for nutrients and heavy metals. Multivariate analyses were performed. Total N, P, Cu and Zn, and available P, Cu, Zn and Mn soil concentrations were associated to the use of organic amendments. Heavy metals concentrations were below established thresholds. Available P (Olsen-P) was identified as an indicator of the previously adopted fertilization management and of the potential of P leaching towards deeper soil layers. Regression analyses were performed. A displacement of available P from the uppermost layer (0–0.3 m) occurs in the breakpoint of 86 mg P kg^−1^ soil. Preventative actions might be established from 53 mg P kg^−1^ soil due to the slowdown in P immobilization. Our results reinforce the importance of setting up P threshold soil levels for best practices of fertilization, as a basis for sustainable agriculture intensification.

**Supplementary Information:**

The online version contains supplementary material available at 10.1007/s10661-023-11901-7.

## Introduction

In a circular economy framework (Ghisellini et al., 2016; Stamm et al., 2022), manures and other organic waste products are valuable resources in agriculture. Their positive effect has been widely studied (Chowdhury & Zhang, 2021; Johnston & Poulton, 2019; Oenema, 2015; Powlson et al., 2012). They increase crop yields by providing nutrients (Ovejero et al., 2016; Perramon et al., 2018) and improve soil physical and biological properties (Domingo-Olivé et al., 2016; Risberg et al., 2017; Valdez et al., 2020; Yagüe et al., 2016). However, other studies relate them with negative impacts on soil health because of the danger of the undesirable increases in heavy metals or nutrients and further water pollution and eutrophication (Alloway & Jackson, 1991; Bomans et al., 2005; Iglesias et al., 2018; Peyraud & MacLeod, 2020; Yagüe & Quílez, 2010).

In the European Union, in order to prevent such environmental impacts, regulations regarding maximum nitrogen (N) applications from materials of organic origin, or maximum concentrations of heavy metals in sewage sludges, have been implemented (European Union, 1991; MAPA, 1990). However, there is not a common EU regulation concerning the amount of P that can be applied in agricultural systems, although restrictions to minimise P losses and ensure best agricultural practices exist in some countries or EU regions (Amery & Schoumans, 2014; Barreau et al., 2018; Rosemarin et al., 2021). The origin of P surplus in agricultural areas is the traditional low recovery by the crop (following application) of the applied mineral P. Phosphorus recovery is close to 16% in world cereal production (Dhillon et al., 2017). When using animal feces, P surplus is also a common trend (Perramon et al., 2018) due to their N/P ratio being lower than the N/P ratio in cultivated plants (Greenwood et al., 2008).

The potential risk of soil P being lost to water bodies is primarily attributed to erosion and runoff (Hughes et al., 2000; Pote et al., 1996; Schröder et al., 2010; Sharpley & Halvorson, 1994). However, the transport of P in soils is a matter of concern as some authors underline the potential risk of losses by leaching (Hooda et al., 1999; Maguire & Sims, 2002; Sims et al., 1998). Phosphorus leaching varies according to soil properties (Jalali & Jalali, 2017; Jarvis, 2007; Liu et al., 2012) and with soil/crop management (McDowell et al., 2001; Svanbäck et al., 2014). The main factors that control the transfer of P from soil to water are the total amount of desorbable P (i.e., that capable of being released from soil to water), the partition between adsorbed and precipitated P forms in the soil solid phase, and the kinetics of phosphate desorption and metal phosphates dissolution (Horta & Torrent, 2007). In calcareous soils, phosphate is adsorbed on calcium carbonate surfaces, although the energy of adsorption is lower than that on surfaces of hydrous oxides. Adsorbed ion cluster and nucleation of calcium phosphate crystals follow. Dicalcium phosphate is the major initial compound precipitated, but some months are required for the formation of less soluble compounds such as octacalcium phosphate (Hagin & Tucker, 1982). Thus, a relatively prolonged availability of phosphates exists. The duration of contact and the soil:solution ratio are additional key aspects (Torrent & Delgado, 2001) and they can be high in percolating drainage water (contact times on the day to week scale). In semiarid areas under irrigation, water percolation occurs and may be useful as it prevents salinization or controls soil salinity levels (Herrero & Castañeda, 2018). Accordingly, the risk of P leaching is also present in irrigated calcareous soils (Beauchemin et al., 1998; Robbins & Smith, 1977; Schoumans et al., 2015). The risk increases in high livestock density areas with an over-use of manure applications exceeding crop needs (Einarsson et al., 2020; Pote et al., 1996; Roswall et al., 2021; Sims et al., 1998; Tóth et al., 2014).

Catalonia (NE Spain) is one of the top regions in the EU for livestock farming (Eurostat, 2021a). Manure or slurry application is the main P input on agricultural land, where 47% of the total P applied comes from livestock. The calculated surplus for this region is about 20.1 kg P ha^−1^, a value substantially higher than the national average of 4.6 kg P ha^−1^ (MAPA, 2023). These figures place Catalonia as one of the EU regions with a relatively high P-surplus (Eurostat, 2021b). In this context, an infraction threshold of 150 mg P kg^−1^ of available P (Olsen-P) has been set in this Spanish region (Generalitat de Catalunya, 2019). Agricultural areas in Catalonia can be employed as pilot areas in the context of the EU strategy *From Farm to Fork* (COM, 2020) as a key initiative of the European Green Deal (COM, 2019). This strategy addresses the use of fertilizers and states ambitious objectives to reduce by half, nutrient losses (as a minimum threshold), and to diminish the use of fertilizers by 20%.

There are various methods to evaluate P in soils, based on different purposes (agronomic and/or environmental). They are also applied at different scales (experimental, local, regional, national). The use of chemical methods to analyze P based on single extractions has been developed worldwide (Renneson et al., 2016); a combination of them might be also used (Sharpley et al., 1996; Wang et al., 2010). Although P leaching has been studied since the 1970s (Logan & McLean, 1973; Novak et al., 1975), the concept of the change point was introduced in the 1990s (Heckrath et al., 1995; Sims et al., 1998). However, more information at real-world farm scale is required, especially from calcareous areas associated with intensive livestock farming, in order to quantify the real risks for water bodies.

Our hypothesis is that in highly calcareous soils under irrigation, former and current mineral and organic fertilization might be characterized by different soil changes in nutrient concentrations and/or in heavy metal soil contents. An important impact of organic fertilization will be associated with the increase of available P (AvP) in the soil upper layer to a given threshold, above which, phosphorus will move in depth, despite the calcareous nature of the soil.

The objectives of the research carried out were as follows: (i) to detect indicators of fertility management according to the farming system and (ii) on the basis of prior results, to assess potential environmental risks of phosphorus leaching in calcareous soils.

This study focuses on a new and modern irrigated area, under an additional high pressure of animal husbandry, as an example (pilot area) to evaluate the performance of former and current fertilization practices on nutrient and heavy metal levels in farmers’ fields and to provide guidance on forthcoming nutrient constraints or challenges.

## Materials and Methods

### Survey Area

Soil fertility assessments were carried out in an agricultural area located in the Ebro river basin (NE of Spain). The climate of the area is dry continental Mediterranean, characterized by hot summers (average of 23–25 °C), cold winters (average of 3–5 °C), and a low annual rainfall (average of 400–550 mm). Soils in that region were developed over detritic and terrigenous materials on the residual platforms of some alluvial fans originated in the area. The greater part of them (Ascaso et al., 1991) are deep soils, calcareous, with a high presence of gypsum. Loam texture predominates with illite as the dominant clay mineral. They are mainly classified as Typic Calcixerept (Soil Survey Staff, 2014).

The study focuses on a recently developed irrigated area (mainly under sprinkler irrigation) which has been in operation from 1999. Water comes from the Algerri-Balaguer channel (41° 49′ 46.38″ N, 0° 34′ 45.48″ E as initial point). Grain cereals, mainly included in a double annual crop rotation of maize and barley, are the most common crops. The municipalities included in the irrigated area sustain an important livestock load (Generalitat de Catalunya, 2019), with pig farming being the most common animal rearing enterprise in the area.

**Fig. 1:**
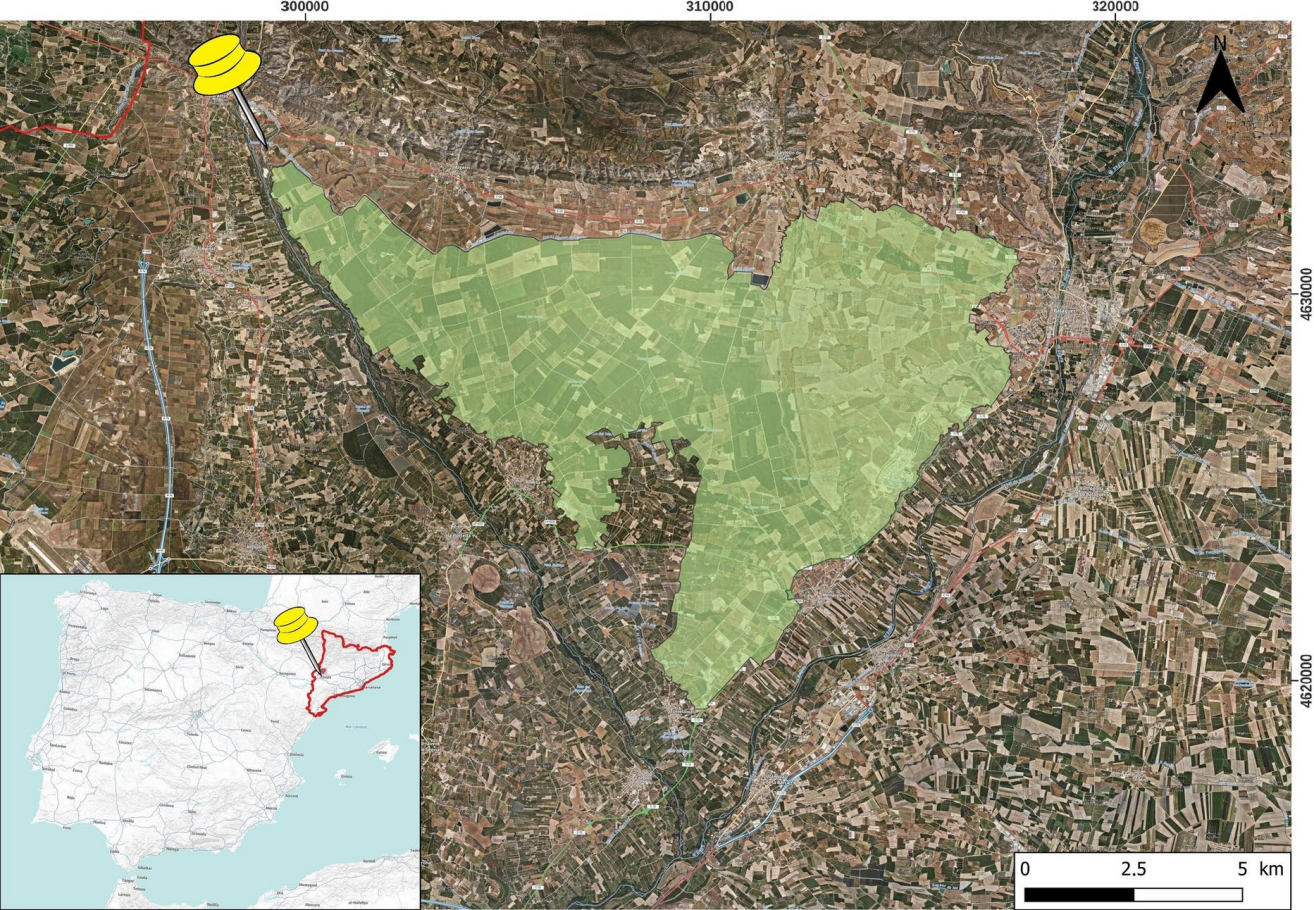
Soil survey area irrigated from the Algerri-Balaguer channel in NE of Spain

The central part of the irrigated Algerri-Balaguer area, between the rivers Noguera Ribagorzana and Farfanya, was chosen (Fig. 1). The studied area covered 733 ha owned by thirty-five people.

Agricultural management information was collected. The questionnaire used included aspects about their main income, livestock business, the use of a service company to apply fertilizers, and the type of fertilization: mineral or organic.

Agriculture in the area was the main source of income (76%). Four different farming systems were identified: crop farming–mineral fertilizer system, crop-livestock farming system, crop farming system, and undefined farming system. The crop farming–mineral fertilizer system is based on mineral fertilization, and farmers do not use manures or any organic amendment. It includes 17% of landowners. At sowing, they mainly use blended mineral fertilizers produced from basic primary fertilizer products such as urea, mono-ammonium phosphate, and potassium chloride. At topdressing, farmers use ammonium nitrate-urea 32 solution or ammonium nitrosulphate. The crop-livestock farming system includes an animal husbandry activity, mainly swine livestock. Slurries and manures produced are reused as fertilizers in the fields owned, and in the surrounding areas, some synthetic N fertilizers might also be applied, sporadically, at maize topdressing. It includes *c*. 30% of the landowners. The crop farming system includes organic fertilizers as a primary nutrient source, mainly due to the high pressure of livestock in the region. However, organic fertilizers are usually combined with synthetic N fertilizers, mainly at topdressing. The undefined farming system includes a group of landowners who are not directly involved in land management decisions. Thus, they do not really have records of fertilization practices as they temporarily lease the land to different farming companies.

The contracting of agricultural services is a usual practice in the area (86%), mainly for harvesting tasks and the application of mineral fertilizers, manures, or pesticides. Regarding fertilizer assessment, a large proportion of respondents (90%) expressed no need for technicians or extension services. However, in Spain, during the period from September 2024 up to September 2025, fertilization assessment will be introduced as compulsory (MPR, 2022).

### Soil Sampling and Analyses

The first survey was conducted in 2014. From the defined area of 733 ha (Fig. 1), fields from 4 up to 8 ha were identified to ensure uniformity in agricultural practices. Composite soil samples included eight sub-samples for every four hectares and a total of 93 composite samples for a depth of 0–0.3 m were obtained. Soil samples were taken before winter sowings. Soil chemical parameters were analyzed: pH (1:2.5 soil:distilled water; potentiometry), electrical conductivity (EC 1:5 soil:distilled water; conductimetry), calcium carbonate equivalent (CCE; Bernard calcimeter), organic carbon (OC; Walkley & Black, 1934; Yeomans & Bremner, 1988), AvP (Olsen-P method; MAPA, 1994a), and available K (AvK; ammonium acetate extraction 1N, pH = 7; MAPA, 1994b). Soil texture (USDA) was characterized using the pipette method in 77 samples (Porta et al., 1986) and the rest (16 samples) were assigned following field methodology (Porta-Casanellas & López-Acevedo Reguerín, 2005).

In 2017, in order to go deeper into P availability and potential displacement from the topsoil (0–0.3 m) to deeper layers (0.3–0.6 m), thirty fields of the different fertilization practices in the area (excluding undefined systems) were sampled once again (60 samples) for AvP and total soil P content (TP). Sampled plots were also chosen in order to cover the amplitude of previous data on AvP. Total P content was analyzed from microwave digested samples (AENOR, 2012) with aqua regia (3:1, v:v, HCl:HNO_3_) and it was quantified using inductively coupled plasma mass spectrometry (UNE-EN 16171; AENOR, 2016). From the upper layer samples, thirteen samples were selected for complementary analyses to detect other potential soil changes in nutrient and heavy metals content that could be related to fertilization management. Total N (TN) was analyzed by the Kjeldahl method (MAPA, 1994c). The total soil content of micronutrients (boron (B), Cu, iron (Fe), Mn, nickel (Ni), and Zn), and heavy metals (cadmium (Cd), cobalt (Co), chrome (Cr), mercury (Hg) and lead (Pb)) was quantified following the same procedure as in TP. The available Zn (AvZn), Mn (AvMn), and Cu (AvCu) were extracted with a DTPA (diethylenetriaminepentaacetic acid) solution (1:2, w:v) following Baker and Amacher (1982).

### Statistical Analysis

Three rounds of statistical analysis were carried out with data from fields belonging to different farming systems. From the initial data of pH, EC, CCE, OC, AvP, and AvK (2014 sampling), a principal component analysis (PCA) was performed (Table A.2). The PCA was also performed with samples (2017 sampling) including additional nutrient and heavy metal data (Table A.3). The number of components was determined at the point beyond which the remaining eigenvalues were relatively small and of comparable size (Jolliffe, 2002; Peres-Neto et al., 2005). The parallel analysis for component retention (Horn, 1965) was also performed (Fig. A.1 and A.2). From such an analysis, screen plots were generated using the Psych package of the R statistical package. Figures were also obtained from the InfoStat package (Di Rienzo et al., 2020).

**Fig. 2:**
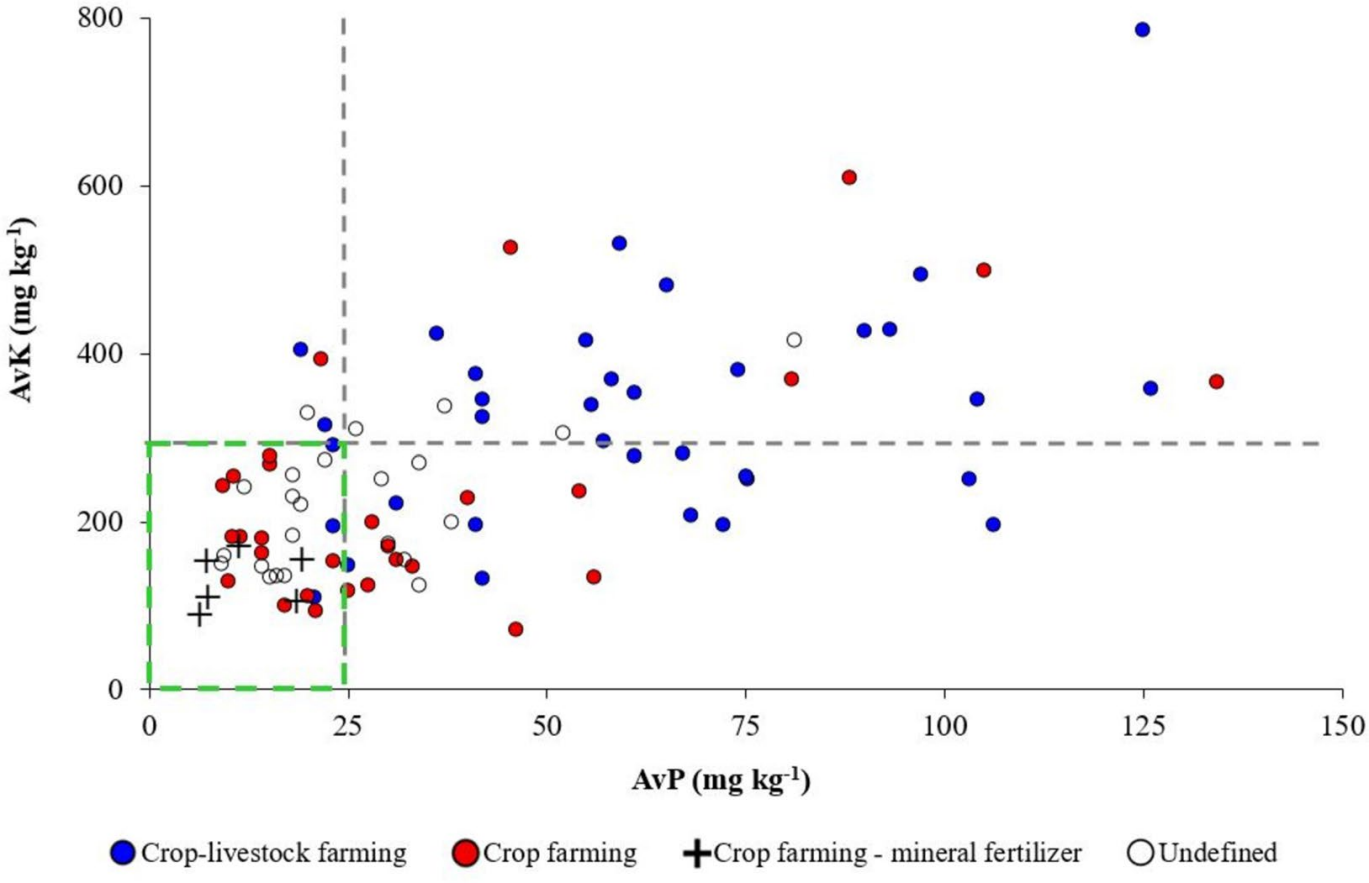
Dispersion of available P (AvP, Olsen-P method) and K (AvK, ammonium acetate extraction) from 93 topsoil samples (0–0.3 m). The vertical and horizontal grey dotted lines represent the warning agronomic threshold of yield non-response for AvP and AvK soil contents, respectively. The left-lower quadrant in green shows the area with no excess of both nutrients according to Martínez and Andrades (2022)

From the second data set of 60 samples (2017 data), nonlinear models were used to describe the P threshold for P displacement in soil from the superficial layer (30 samples) to the deeper layer (30 samples). A piecewise regression or segmented regression was used to identify natural breakpoint between the variables. The relationship between AvP and TP (0–0.3 m) was also established. The models holding the best fit to the data, based on the Akaike Information Criterion (AIC), were chosen. In order to ensure the validity of the performed statistical analysis, the assumptions underlying the model were rigorously assessed. The following key assumptions were examined: independence of observations, constant variance (homoscedasticity), normality of residuals, and appropriateness of the random effects specification. From such an analysis, screen plots were generated using the Psych package of the R statistical package and a Durbin-Watson test was done. The AgroReg library (Shimizu & Gonçalves, 2022, 2023) was employed within the R software environment (R Core Team, 2023).

## Results

### Soil Fertility

Soils of sampled fields matched general soil properties of the area (Table A.1). They were calcareous (the average of CCE was 254 g kg^−1^) and the pH average was 8.1 (± 0.3). The electrical conductivity (median of 0.31 dS m^−1^) agreed with the gypsum presence, but it also provided an alert about potential restrictions to crop development in some fields. Soils had a low OC content (average of 12 g kg^−1^). The texture was mainly loam although one sample was sandy loam, seven were clay loam, and ten were silty loam.

**Table 1:** Descriptive statistics of total N (TN), available P (AvP), available Cu (AvCu), available Mn (AvMn), available Zn (AvZn), and total soil content of P, B, Cu, Fe, Mn, Ni, Zn, Cd, Co, Cr, Hg, and Pb in thirteen samples (0–0.3 m). Official upper threshold values^a^ for some variables, and for soils with a pH ≥ 7, are included

Variable	TN	TP	AvP	AvCu	AvMn	AvZn	B	Cu	Fe	Mn	Ni	Zn	Cd	Co	Cr	Hg	Pb
	(g kg^−1^)						(mg kg^1^)										
Mean	1.5	1044	73	2.9	13.3	9.0	17	27	19,298	448	19.9	90.2	0.12	7.8	24	0.21	12
Std. deviation	0.4	278	43	1.6	8.7	2.2	3	6	2686	55	2.8	28.0	0.06	1.0	3	0.15	2
Percentile 25th	1.2	892	35	1.7	9.3	7.5	14	22	18,364	416	18.6	68.7	0.07	7.3	22	0.12	11
Median	1.5	1048	86	3.1	12.9	9.5	17	29	20,066	453	19.2	88.4	0.13	7.9	23	0.16	13
Percentile 75th	1.7	1221	101	4.4	22.3	9.9	19	31	20,590	489	21.9	114.8	0.17	8.4	27	0.25	14
MAPA (1990)^a^								210	-	-	112	450	3.0	-	150	1.5	300
GC (2019)^a^			80–150														
MPR (2022)^a^								100			70	200	1.5			1.0	100

^a^*MAPA* Spanish Ministry of Agriculture, Fisheries and Food *GC* Generalitat de Catalunya, Catalan Government, Spain *MPR* Spanish Ministry of the Presidency, Relations with Parliament and Democratic Memory

Soil fertility (0–0.3 m) showed a wide range of values in AvP and AvK, from deficiency to yield non-response (Fig. 2).

**Fig. 3:**
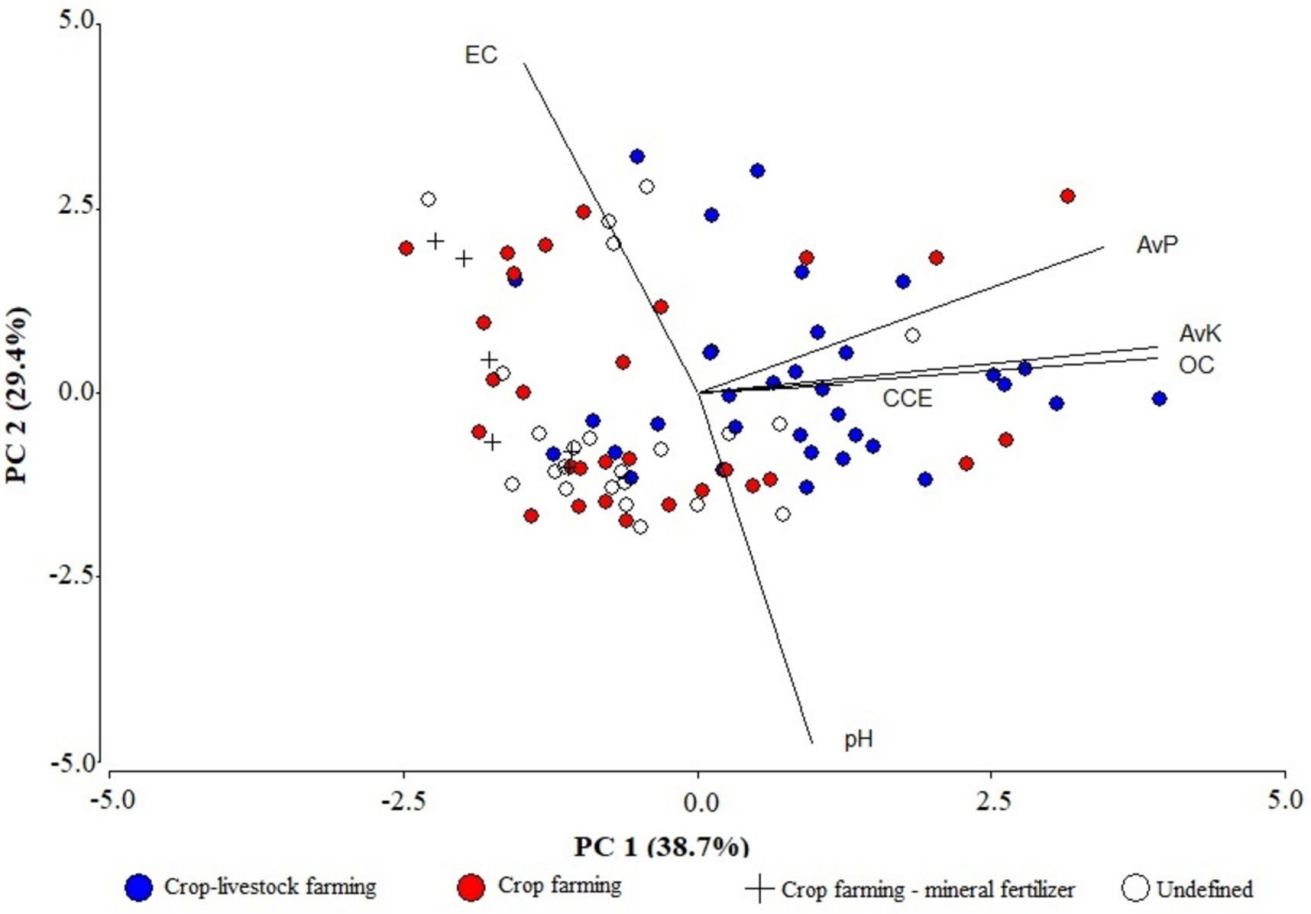
Principal component analysis biplot (PC1 vs. PC2) of the 6 studied variables according to the fertilization management. Letters on the figure are associated with soil chemical parameters: pH, electrical conductivity 1:5 (EC), organic carbon (OC), calcium carbonate equivalent (CCE), available P (AvP), and available K (AvK)

Principal component analysis indicated that crop-livestock agricultural system was mainly associated with high AvP and AvK values, while crop farming–mineral fertilizer was associated with low ones (Fig. 3). Crop farming–mineral fertilizer was also associated with the lowest EC values. The parallel analysis also indicated two dimensions to retain (Fig. A.1).

In the second and more oriented soil analysis (2017 data), none of the samples exceeded the threshold limits for micronutrients or heavy metals (Table 1). However, about half of the samples exceeded the warning level of 80 mg P kg^−1^ for AvP for this region (Generalitat de Catalunya, 2019).

**Fig. 4:**
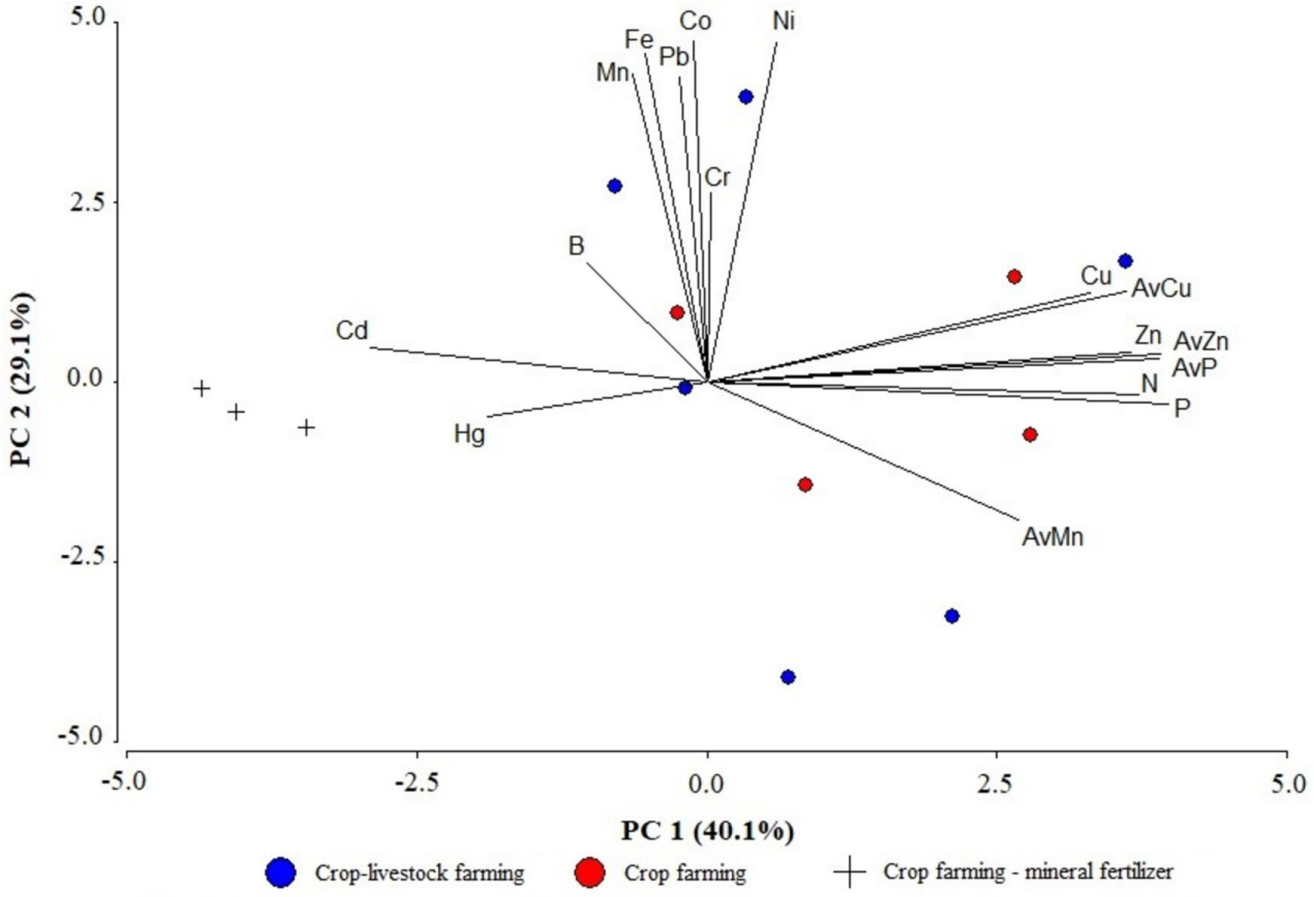
Principal component analysis biplot of the 17 studied variables according to the fertilization management. Letters on the figure are associated with available (Av) or total soil content of different elements

The second PCA analysis showed that five factors were able to explain almost 93% of the variability, and that just two components explained close to 70% (Table A.3). The parallel analysis (Fig. A.2) signalled two components for this data set. The PC1 explained 40.1% of the variability (Fig. 4). The use of mineral fertilizers was also represented by PC1, but as antagonist to the first mentioned group.

Mineral fertilizers use might be characterized by the Cd levels in soil, although they were below (× 10) of the threshold levels (Table 1 and Fig. 4). The rest of the total elements analyzed (e.g., Cr, Fe, Mn, Ni, Pb) were associated with the second component (PC2), which explained 29.1% of the variability and can be related to soil background (edaphogenesis).

### Phosphorus Mobility

**Fig. 5:**
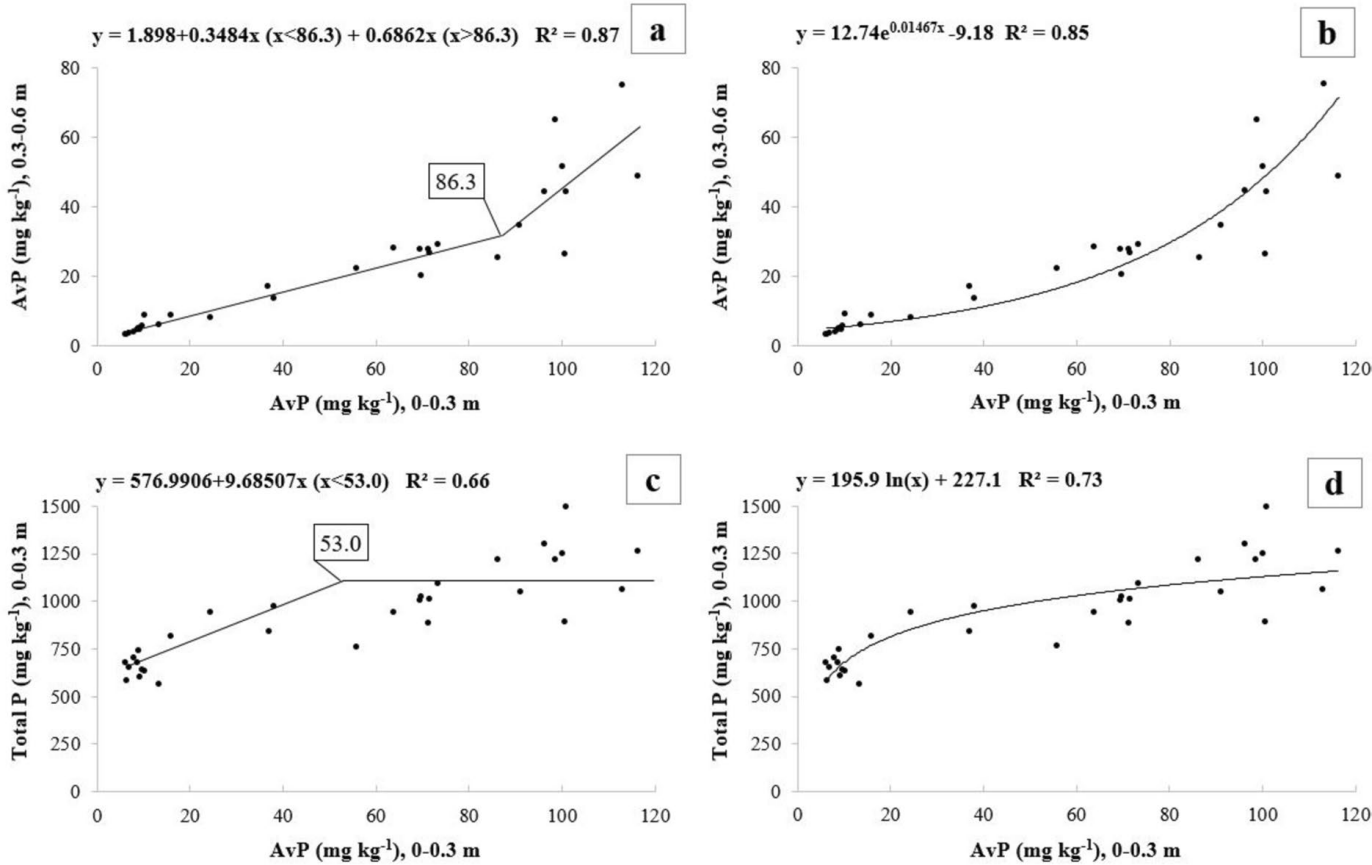
Phosphorous displacement behavior in calcareous soils (*n* = 30). Available P content (mg kg^−1^) at the superficial layer (0–0.3 m) versus the one at the next deeper soil layer (0.3–0.6 m) according to **a** split-line model or **b** exponential model and available P content (mg kg^−1^) versus total P content (mg kg^−1^), both at 0–0.3 m, and according to **c** lineal plateau model and **d** logarithmic model

The AvP values decreased with soil depth (Fig. 5a–b). The relationship of AvP between the two soil layers (0–0.3 m vs. 0.3–0.6 m) followed a significant split-line model (*R*^2^ = 0.87, AIC = 209) (Fig. 5a) with a breakpoint at 86 mg P kg^−1^. The above model had a better fit than the exponential one (*R*^2^ = 0.85, AIC = 211) (Fig. 5b).

When AvP values from the topsoil (0–0.3 m) were compared with the TP ones at the same depth, a positive relationship was obtained (Fig. 5c–d). The lineal plateau model showed an acceptable correlation (*R*^2^ = 0.66) between both variables (AIC = 390), with a break slope point close to the AvP value of 53 mg P kg^−1^ (Fig. 5c). However, the logarithmic model had a better fit (*R*^2^ = 0.73, AIC = 381). It also indicated that P immobilization, at 0–0.3 m, slowed down beyond 50–60 mg kg^−1^ of AvP (Fig. 5d).

## Discussion

### Soil Fertility According to the Farming System

General data of soil pH, EC and CCE (Table A.1) are related to the intrinsic soil conditions of the area. The pH and the CCE were both in accordance with the soil types described in the soil map (1:25,000) of Catalonia (Ascaso et al., 1991). High EC values are based in the presence of gypsum in the region (Herrero et al., 1996). Despite the tendency to increase soluble salts when fertilization is not based on mineral fertilizers (Fig. 3), several authors (Yagüe & Quílez, 2013; Bosch-Serra et al., 2020) have signalled the low relevance of EC changes with manure at high sustained rates.

The dispersion graph of AvP and AvK (Fig. 2) allowed an initial differentiation of the farming systems identified in the survey, which was confirmed by the PCA analysis (Fig. 3, A.1 and Table A.2). Most of the farms which combine animal husbandry with crop farming (named as crop-livestock farming system) are on the upper right quadrant side. The above means that more than half and one third of sampled plots exceeded, respectively, the thresholds of 25 mg P kg^−1^ and 295 mg K kg^−1^ for yield non-response (Fig. 2) that have been established by Martínez and Andrades (2022) for irrigated farming systems with a loamy soil texture.

The observed high P and K levels (Fig. 2) are in accordance with reports from different authors on using organic fertilizers (Bosch-Serra et al., 2020; Chowdhury & Zhang, 2021; Johnston & Poulton, 2019; Oenema, 2015; Powlson et al., 2012), although N criteria could have been followed for manure rate adjustment. By contrast, farmers without animal husbandry activities, mainly the ones who only apply mineral fertilizers (crop farming–mineral fertilizer system), are clearly distributed on the lower left quadrant. The above group shows low P and K concentrations (*c*. 6–7 mg Olsen-P kg^−1^, 90–100 mg K kg^−1^), even close to deficiency levels for P (< 7 mg kg^−1^) and K (< 80 mg K kg^−1^) established for loamy soils by Rodríguez Martín et al. (2009) and MARM (2010). In fact, farmers using mineral fertilizers tended to better adjust P needs for crop development (Goss et al., 2013). In the established pilot area, the above means that around 20 and 30% of the sampled plots showed a constraint (insufficient content) on AvP (< 13 mg kg^−1^) and on AvK (< 160 mg kg^−1^). Some landowners ought to build up their AvP and AvK soil contents from deficiency to maintenance levels to avoid unwanted soil and plant side-effects. Crop farming systems or undefined farming systems, as they apply (or they can eventually apply) manures or slurries, showed some dispersion although most of the samples were below the established thresholds (Fig. 2). The unbalanced soil fertility in some fields means that there is a real possibility to improve fertilization management and even to reinforce the nutrient circular economy in the area.

After this first approach, the use of AvP as a potential indicator of the fertilization management on calcareous soils was reinforced. It is corroborated in the second APC analysis (Table A.3 and Fig A.2). Although AvK could also have been taken into account, it was not prioritized because of the higher needs of the crops and the potential interference from other management practices (e.g., export or incorporation of straw), which might influence the K net increase (Zhang et al., 2021).

Availability of Cu, Mn, and Zn is constrained by high pH (Ballabio et al., 2018; Mousavi et al., 2013). The application of manures should help to increase their plant availability (Fig. 4) and to reduce the costs from mineral fertilization supplies (Ballabio et al., 2018; Gómez-Miguel & Sotés, 2014; Rodríguez-Martín et al., 2009). In fact, intensive livestock feeding introduces essential elements (e.g., Cu and Zn) in animal diets, in which they may be used as growth promoters or because of their medical properties, among other characteristics (Bernhoft et al., 2014; Poulsen, 1998). This common practice has driven up the relevant content of such elements in the manure, so they become ultimately present in agricultural soils after their application (Mantovi et al., 2003; Delgado et al., 2014). This consequence was observed in our study (Fig. 4) in concordance with other authors (Berenguer et al., 2008). It is important to underline that the results obtained (Table 1) are far below the thresholds established in the current Spanish legislation (MAPA, 1990; MPR, 2022). Furthermore, the average of total Mn content (448 mg kg^−1^) is close to the data obtained by Gómez-Miguel and Sotés (2014) in Spanish vineyard soils and to that obtained by Bosch-Serra et al. (2020) with dairy cattle slurries (334 mg kg^−1^). Total Mn content is influenced by its lithological origin (Foregs, 2008) but AvMn (Fig. 4) might be affected by the questionable high supplementary levels added in pig diets (Kerkaert et al., 2021).

Cadmium content from mineral fertilizers (phosphate fertilizers) has been associated with soil pollution (de Vries et al., 2022; Kubier et al., 2019; Römkens et al., 2018). It has been under discussion during the implementation of the new EU fertilizers regulation (The Council of European Communities, 2019). Nevertheless, this matter has concerned the EU since the 1990s, when some member states set up national regulations on this element (Bomans et al., 2005). In our study, the use of mineral fertilization was associated with total Cd soil content (Fig. 4), although such results were very much lower than the current legislation thresholds (MAPA, 1990; MPR, 2022). There is not sufficient information about the mineral fertilization history of the sampled soils, but the Cd availability in calcareous soils might be a matter of future interest.

The rest of the soil microelements and heavy metals analyzed showed no association with fertilization practices (Fig. 4), and they were probably related to edaphogenesis. The average contents of the trace elements analyzed were similar to previous results obtained in the area from a Spanish survey (López-Arias & Grau-Corbí, 2004).

### Phosphorus Vertical Displacement in Calcareous Soils

In this research, AvP soil content increased with organic P supplies (Fig. 3) and in both soil layers (Fig. 5a, b). The AvP at 0.3–0.6 m rose up *c*. 7 times over the mineral background level. This result agrees with Sharifi et al. (2020) who described the downward movement of orthophosphate-P and soluble organic P to higher soil depths in the locations with high chance of excretion/urination in a paddock. It also agrees with other authors (James et al., 1996; McDowell & Sharpley, 2001; Sims et al., 1998) who also reported, on manured soils, TP and AvP accumulations in depth.

The situation described raised the need to identify which calcareous soils might be considered at risk due to the P release (desorption) from soil to water. Some approaches might be similar to the ones developed in acid soils (e.g., the P-saturation degree defined for P leaching in sandy soils (Bomans et al., 2005) or the Olsen-P threshold values for desorption of P from soil (solid phase) to water and according to soil:solution ratio (Horta & Torrent, 2007)). It should be pointed out that at comparable Olsen-P values, the acid soils release less phosphate to water than the calcareous soils (Horta & Torrent, 2007).

According to the data obtained in calcareous soils, the amount of AvP in the topsoil warns of the risk of P depth shift, regardless of any particular P desorption scenario. Data indicate a rapid increase in the accumulation of AvP at 0.3–0.6 m; when in the upper layer Olsen-P concentration, it is above 86.3 mg kg^−1^ (Fig. 5a). This finding is supported by the non-linear accumulation of TP at 0–0.3 m (Fig. 5c). Once the topsoil approaches to within a certain degree of its limit capacity for P sorption (adsorption or precipitation), and the soil:solution ratio decreases, a concomitant and significant P enrichment of soil solution (Horta et al., 2013) can lead to P transfer to deeper layers or to drainage water. Besides, a movement of soluble organic P via dissolved OC or colloidal particles may occur (Chardon et al., 1997; James et al., 1996) through macropores (Freiberger et al., 2014; Jarvis, 2007). Macroporosity is at the same time favored by organic fertilization (Valdez-Ibáñez et al., 2019; Mateo-Marín et al., 2021). Once the subsoil is enriched, P mineralization could release P (James et al., 1996) but P can also be sorbed (Horta et al., 2013) which, in the short term, limits P transfer to drainage water.

As this research was implemented at farm scale, and as a pilot area, an additional and conservative warning change interval might be considered, according to changes in TP following a logarithmic model (Fig. 5d). Under the calcareous conditions of our study, soils with AvP content between the range of 53–86 mg kg^−1^ (Fig. 5) are located at the starting point of a warning situation in TP increase (P immobilization in the upper layer), although the breakpoint for P depth shift is defined at 86 mg kg^−1^. The established warning interval for calcareous soils is in line with Heckrath et al. (1995) in UK and Jalali and Jalali (2017) in Iran, working on soils of slightly lower pH (pH average of 7.5 ± 0.2). They established an AvP (Olsen-P) change point of 60 mg P kg^−1^ and 61.5 mg P kg^−1^, respectively, and above which P concentration in drainage water increases rapidly. Our warning interval is also in line with the warning threshold of 80 mg P kg^−1^ established for AvP (Olsen-P) in the Spanish region of Catalonia (Generalitat de Catalunya, 2019).

The reduction of P surplus should be achieved by considering some basic rules such as the use of soil analysis to try to take advantage of residual AvP (Schröder et al., 2011) and to match P crop needs with P inputs (Hooda et al., 2001). Our data showed a safety interval between the agronomic threshold (above which there is no yield response to AvP (> 25 mg kg^−1^, Olsen-P), the changing zone that constraints TP increase in the upper layer (53–86 mg kg^−1^, Olsen-P) and the breakpoint (86 mg kg^−1^, Olsen-P) for vertical AvP displacement.

## Conclusions

In irrigated calcareous soils, AvP (Olsen-P) is a useful indicator of differences in fertilization management between farming systems. In the studied area, differences in fertilization management did not increase warnings about the potential raising of heavy metal concentrations in soils.

An assessment of available P also provides an alert about potential environmental impacts, as it is an indicator of the risk of P displacement from the topsoil (0–0.3 m) to deeper layers. A breakpoint for the assessment of AvP leaching risk (from a soil layer) was observed at 86 mg Olsen-P kg^−1^. A warning scenario, from 53 mg Olsen-P kg^−1^, could be established based on the decrease (following a logarithm model) of TP concentration in the upper layer.

The results of our study can establish the basis for complementary measures to minimize the build-up of P in soil through a better adjustment of organic fertilizer rates. They also support a nutrient circular economy schedule in areas with an important and intensive animal husbandry activity. In farming systems with a high risk of P leaching, these measures will prevent P losses and ensure better environmental protection.

### Supplementary Information

Below is the link to the electronic supplementary material.

Supplementary file1 (DOCX 125 KB)

## Data Availability

The datasets generated during and/or analyzed during the current study are available from Dr Carlos Ortiz on reasonable request.

## Declarations

**Ethical Approval**

Not applicable.
