# Supplementary material for: Soil Fertility and Phosphorus Leaching in Irrigated Calcareous Soils of the Mediterranean Region
Source: Environ Monit Assess. 2023 Oct 26;195(11):1376. doi: 10.1007/s10661-023-11901-7 (PMC10602959; doi:10.1007/s10661-023-11901-7)
Supplement: Supplementary file 1 — Supplementary file1 (DOCX 125 KB) [file 10661_2023_11901_MOESM1_ESM.docx]

**Soil fertility and phosphorus lixiviation in irrigated calcareous soils of the Mediterranean region**

Carlos Ortiz^a,b^; Silvia Pierotti^c^; M. Gabriela Molina^c^ ; Àngela D. Bosch-Serra^a,*^

^a^ Department of Chemistry, Physics, Environmental Sciences and Soil, University of Lleida, Avda Alcalde Rovira Roure 191, E-25198, Lleida, Spain. Tel: +34 973 702899 Fax: +34 973 702613.

^b^ DACC, Department of Climate Action, Food and Rural Agenda, Generalitat de Catalunya, Avda Alcalde Rovira Roure 191, E–25198 Lleida, Spain

^c^ Cátedras de Bioestadística I y II. Facultad de Ciencias Exactas Físicas y Naturales, Universidad Nacional de Córdoba, Córdoba, Argentina.

**SUPPLEMENTAL MATERIAL**

**Table A.1** Descriptive statistics for chemical parameters of 93 soil samples (0–0.3 m depth): pH (1:2.5, soil:water), electrical conductivity (EC, 1:5 soil:water), calcium carbonate equivalent (CCE, Bernard calcimeter) and organic carbon (OC, Walkley-Black method).

|  | **pH** | **EC**  (dS m^-1^) | **CCE**  (g kg^-1^) | **OC**  (g kg^-1^) |
| --- | --- | --- | --- | --- |
| Mean | 8.1 | 0.7 | 254 | 12 |
| Standard deviation | 0.2 | 0.7 | 84 | 3 |
| Percentile 25^th^ | 8.0 | 0.2 | 240 | 10 |
| Median | 8.2 | 0.3 | 260 | 11 |
| Percentile 75^th^ | 8.3 | 0.8 | 310 | 13 |

**Table A.2** Principal component analysis of soil (n= 93) main properties (AvP, AvK, EC, CCE. OC, pH) in the first set of soil data from 2014

| Principal Component | Eigenvalue | Variability (%) | Cumulative (%) |
| --- | --- | --- | --- |
| PC1 | 2.32 | 38.67 | 38.67 |
| PC2 | 1.77 | 29.42 | 68.09 |
| PC3 | 0.98 | 16.32 | 84.42 |
| PC4 | 0.40 | 6.71 | 91.13 |
| PC5 | 0.34 | 5.61 | 96.74 |
| PC6 | 0.20 | 3.26 | 100.00 |


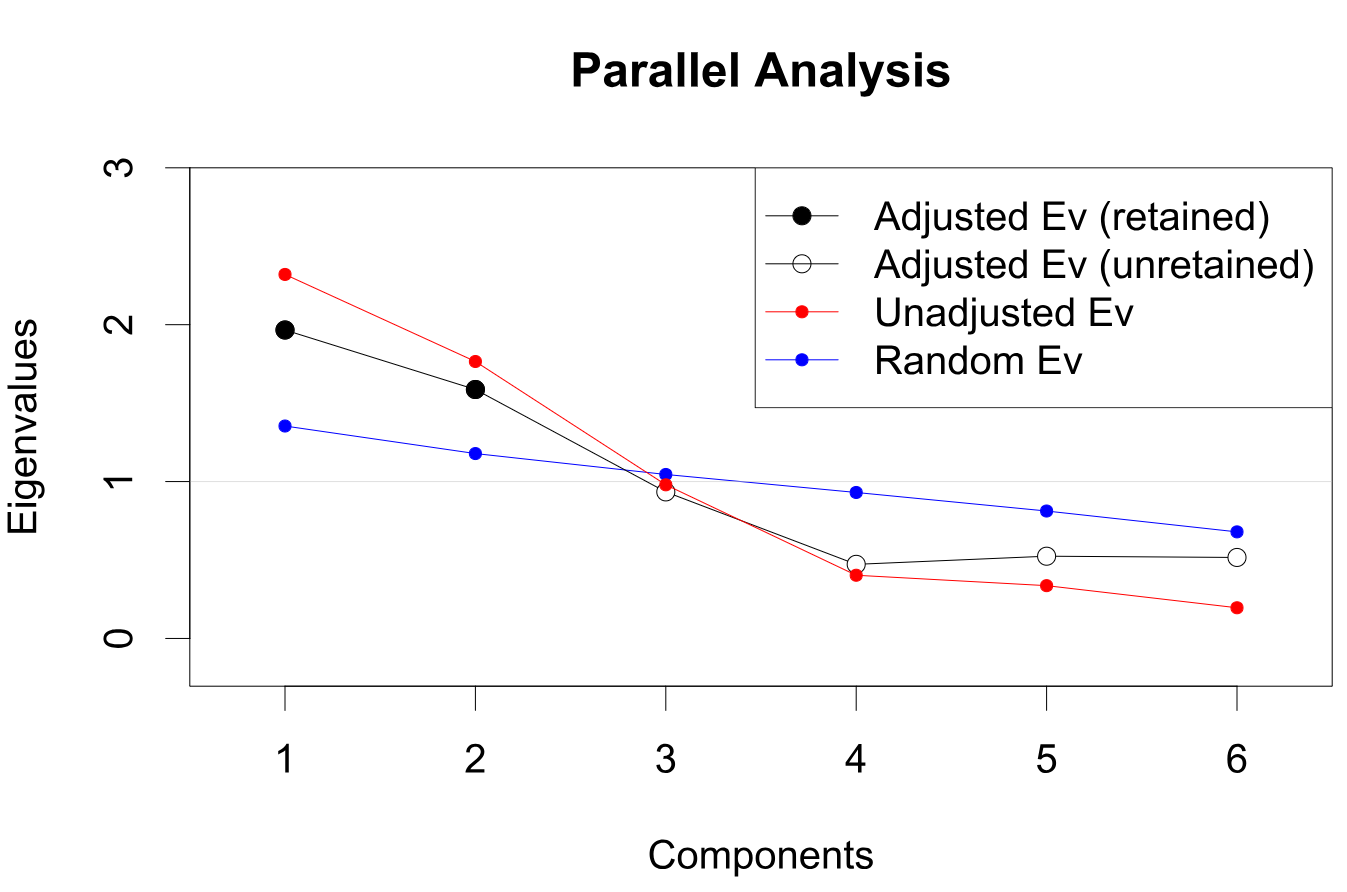


**Fig. A.1.** Parallel analysis of the principal component analysis (Table A.2) on simulated data with two true components underlying six variables. Non-retained components are marked with a hollow circle on the adjusted eigenvalues curve. Note that the cut-off black solid line runs through the elbow of eigenvalue (Ev) actual data (black line).

**Table A.3** Principal component analysis of soil nutrients and heavy metals in the second set of soil data (n=13) from 2017

| Principal Component | Eigenvalue | Variability (%) | Cumulative (%) |
| --- | --- | --- | --- |
| PC1 | 6.8205 | 40.12 | 40.12 |
| PC2 | 4.9551 | 29.15 | 69.27 |
| PC3 | 2.0724 | 12.19 | 81.46 |
| PC4 | 1.1588 | 6.82 | 88.27 |
| PC5 | 0.8676 | 5.10 | 93.38 |


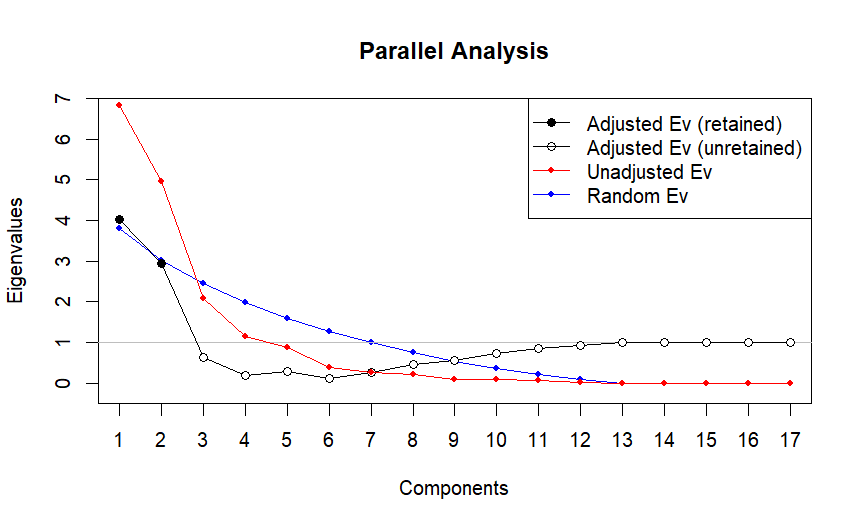


**Fig. A.2** Parallel analysis of the principal component analysis (Table A.3) on simulated data with two true components underlying 17 variables. Non-retained components are marked with a hollow circle on the adjusted eigenvalues curve. Note that the cut-off black solid line runs through the elbow of eigenvalue (Ev) actual data (black line)
